# Gut Microbiome and Organ Fibrosis

**DOI:** 10.3390/nu14020352

**Published:** 2022-01-14

**Authors:** Carolina F. F. A. Costa, Benedita Sampaio-Maia, Ricardo Araujo, Diana S. Nascimento, Joana Ferreira-Gomes, Manuel Pestana, Maria J. Azevedo, Ines S. Alencastre

**Affiliations:** 1 i3S—Instituto de Investigação e Inovação em Saúde, Universidade do Porto, 4200-135 Porto, Portugal; carolina.costa@i3s.up.pt (C.F.F.A.C.); benedita.maia@i3s.up.pt (B.S.-M.); ricardo.araujo@i3s.up.pt (R.A.); dsn@ineb.up.pt (D.S.N.); jogomes@med.up.pt (J.F.-G.); manuel.pestana@i3s.up.pt (M.P.); mjazevedo@i3s.up.pt (M.J.A.); 2 INEB—Instituto Nacional de Engenharia Biomédica, Universidade do Porto, 4200-135 Porto, Portugal; 3 ICBAS—Instituto de Ciências Biomédicas Abel Salazar da Universidade do Porto, 4050-313 Porto, Portugal; 4 FMDUP—Faculdade de Medicina Dentária da Universidade do Porto, 4200-393 Porto, Portugal; 5 Departamento de Biomedicina, Faculdade de Medicina da Universidade do Porto, 4200-319 Porto, Portugal; 6 IBMC—Instituto de Biologia Molecular e Celular, Universidade do Porto, 4200-135 Porto, Portugal; 7 Departamento de Medicina, Faculdade de Medicina da Universidade do Porto, 4200-319 Porto, Portugal; 8 Departamento de Nefrologia, Centro Hospitalar Universitário São João, 4200-319 Porto, Portugal; 9 ACTA—Academic Center for Dentistry Amsterdam, University of Amsterdam and Vrije Universiteit, 1081 LA Amsterdam, The Netherlands

**Keywords:** gut microbiome, intestinal fibrosis, liver fibrosis, kidney fibrosis, lung fibrosis, heart fibrosis, diet, therapeutic strategies

## Abstract

Fibrosis is a pathological process associated with most chronic inflammatory diseases. It is defined by an excessive deposition of extracellular matrix proteins and can affect nearly every tissue and organ system in the body. Fibroproliferative diseases, such as intestinal fibrosis, liver cirrhosis, progressive kidney disease and cardiovascular disease, often lead to severe organ damage and are a leading cause of morbidity and mortality worldwide, for which there are currently no effective therapies available. In the past decade, a growing body of evidence has highlighted the gut microbiome as a major player in the regulation of the innate and adaptive immune system, with severe implications in the pathogenesis of multiple immune-mediated disorders. Gut microbiota dysbiosis has been associated with the development and progression of fibrotic processes in various organs and is predicted to be a potential therapeutic target for fibrosis management. In this review we summarize the state of the art concerning the crosstalk between intestinal microbiota and organ fibrosis, address the relevance of diet in different fibrotic diseases and discuss gut microbiome-targeted therapeutic approaches that are current being explored.

## 1. Introduction

Fibrosis is a complex pathological process that results from excessive deposition of extracellular matrix (ECM) components in response to tissue injury [1]. It is the final pathological outcome of most chronic inflammatory diseases and a major contributor to organ malfunction and failure [2]. Fibrotic tissue responses can affect nearly every tissue and organ system and are responsible for up to 45% of all deaths in developed countries [3]. Despite being recognized as a major health problem worldwide, very few treatments are currently available for the treatment of fibrotic disorders and they have limited efficacy [3].

Upon tissue injury, an intricate cascade of events aiming the repair of tissue architecture and function takes place. Cell damage leads to the immediate release of a myriad of inflammatory mediators such as growth factors, cytokines and chemokines, which promote leukocyte infiltration, the activation of fibroblasts into myofibroblasts (collagen secreting, *α*-smooth muscle actin (SMA)-expressing fibroblasts) and the synthesis of ECM components. In cases of minor and non–repetitive injury, the increased deposition of ECM components is transient and once the wound is repaired, myofibroblasts undergo apoptosis and the reparative response ceases. When the injury is severe or enduring, however, fibroblast activation persists as a chronic, uncontrolled process and ECM components tend to accumulate continuously, leading to the formation of a permanent fibrotic scar, organ malfunction and ultimately organ failure [1]. Myofibroblasts are the key cellular mediators of the repair process and the major factor responsible for the secretion of most ECM proteins. These cells can result from activation of resident fibroblasts and mesenchymal cells but may also originate from a large variety of different cell types such as cells of the vascular wall, endothelial cells, epithelial cells, fibrocytes (circulating fibroblast-like cells derived from bone-marrow stem cells) and bone-marrow-derived progenitors such as adipogenic progenitors [4]. Myofibroblasts can be activated through several different stimuli, such as paracrine signals from immune cells, autocrine factors secreted by myofibroblasts and pathogen-associated molecular patterns (PAMPS) produced by pathogenic microorganisms that interact with pattern recognition receptors (PPRs, such as toll-like receptors (TLRs)) on fibroblasts [5]. 

The breaking point at which the reparative process is no longer able to cope with the insult and becomes dysregulated, therefore entering in a fibrotic path, is not known. Still, it is broadly accepted that once fibrinogenic pathways are activated, the process enters a vicious cycle where even the structural changes of fibrotic tissues themselves are feeders of a further fibrotic response by regulating the differentiation, recruitment, proliferation and activation of ECM-producing myofibroblasts [6].

Regardless of the initial trigger, the innate and adaptive arms of the immune system have a major role in the onset and progression of the fibrotic response and several different immunoregulatory pathways have been pinpointed [7]. Still, several pieces of evidence point to other immune-independent mechanisms triggering fibrotic processes and that inflammation may even be necessary for the reversion of progressive fibrosis [8,9]. Such evidence sheds some light onto the lack of success of anti-fibrotic therapies targeting inflammatory processes and suggests that different paths should be Investigated.

In recent years, the role of the gut microbiota in fibrotic processes has been gaining increasing interest. The intestinal microbiota is composed of around 100 trillion bacteria of about 1000 different species which, in healthy conditions, maintain a symbiotic relationship with the host, exerting important and complex functions in metabolism and immunity [10].

**Figure 1:**
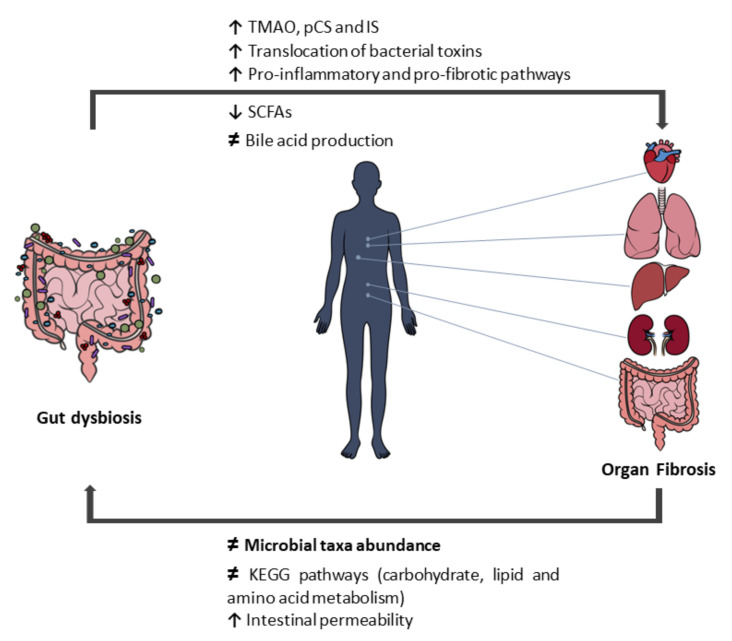
Gut dysbiosis and organ fibrosis. TMAO, trimethylamine N-oxide; pCS, p-cresyl sulfate; IS, indoxyl sulfate; SCFAs, short-chain fatty acids; KEGG, Kyoto Encyclopedia of Genes and Genomes. ↑ increased; ↓ decreased; ≠ altered.

Alterations of the gut bacterial population toward a pathological phenotype—dysbiosis—can result in the accumulation of toxic compounds, namely, uremic toxins, and in the depletion of beneficial products (such as short-chain fatty acids (SCFAs)) [11,12]. The dysbiotic state is often associated with disrupted intestinal barrier integrity, facilitating the translocation of bacteria and bacterial products into circulation, and inducing the systemic activation of immune and inflammatory responses that can directly or indirectly cause tissue damage [13]. In genetically susceptible hosts, a dysregulation of the microbiota–immunity interactions are believed to contribute to the onset and progression of a multitude of immune-mediated disorders [13]. An increasing amount of data has been highlighting gut dysbiosis as a major promotor of disease pathogenesis, representing an intrinsic link with the development of fibrosis on several organs (Figure 1).

The aim of this review is to summarize the role of intestinal microbiota in the development and progression of fibrosis in different organs, such as the intestine, the liver, the kidney, the lungs and the heart, and provide an update on the current strategies targeting the gut microbiome in the treatment of fibrotic diseases.

## 2. Intestinal Fibrosis

Chronic inflammation appears to be the major event triggering gut fibrogenesis, through mesenchymal cell recruitment and activation [14]. Intestinal fibrosis-associated inflammation may occur much more severely than in liver, kidney, or lung fibrosis [15,16], such as that occurring in inflammatory bowel disease (IBD). Although the intestine shows an impressive ability to fully regenerate after short-lived insults (infections, acute peptic ulcer, etc.), under the presence of continuous severe inflammation, the mesenchymal cells (in the form of fibroblasts, myofibroblasts, or smooth muscle cells) are continuously activated, producing excessive ECM, and leading to fistulae or stricture formations with possible intestinal obstruction [15]. To date, there is no pharmacological therapy that is effective in reversing intestinal fibrosis; therefore, a deeper understanding of intestinal fibrosis mechanisms is urgent in the search for innovative strategies.

The understanding of the role of the gut microbiome in the pathogenesis of intestinal fibrosis started to be unraveled in IBD, the most extensive studied disease that is deeply associated with intestinal fibrosis development. IBD includes ulcerative colitis and Crohn’s disease. In Crohn’s disease, intestinal fibrosis is a frequent complication that may lead to bowel wall thickening, strictures and stenosis due to general inflammation, causing the remodeling of the entire intestinal wall, associated with an enhanced deposition of ECM components [17,18]. In ulcerative colitis, the triggering of fibrosis was recently recognized and is associated with colon shortening and stiffening due to the accumulation of ECM in the mucosal and submucosal layers, as well as with inflammation and disruption of the epithelial layer due to tight-junction damage [19,20].

The link between the gut microbiome and intestinal fibrosis was unveiled in IBD, on the one hand, because these patients showed a dysbiotic and pro-inflammatory gut microbiota [21] and, on the other hand, in germ-free animal models of colitis or in Crohn’s disease patients undergoing antibiotherapy, where the gut inflammation was found to be absent or clearly improved [22]. Moreover, adherent-invasive *Escherichia coli* (AIEC), a pathotype of *E. coli*, and *Salmonella enterica* serovar *Typhimurium* (*S. typhimurium*) have been shown to induce inflammation (through elevated T helper (TH) 1 and TH17 immune responses) in IBD animal models, leading to subsequent fibrosis development [23,24,25,26]. In addition, and similarly to what is found in Crohn’s disease patients, extensive ECM deposition was observed in AIEC-infected mice, along with higher expression levels of collagen types I/III, and the enhanced expression of profibrotic mediators such as transforming growth factor-β1 (TGF-β1), connective-tissue growth factor, and insulin-like growth factor I (IGF-I) [26]. In agreement with this, in patients with Crohn’s disease, AIEC strains were associated specifically with ileal mucosa and were suggested to locally trigger the initiation or perpetuation of the inflammatory disease [27].

Currently, some mechanisms and molecules associated with the gut microbiome were already recognized to be involved in the pathogenesis of intestinal fibrosis. The bacterial lipopolysaccharides (LPSs), also known as endotoxins, present in the external structure of the gram-negative bacterial cell wall, are known to promote the profibrotic activation of intestinal fibroblasts, with increased nuclear factor-κB (NF-κB)-light-chain-enhancer of activated B cell promoter activity and collagen contraction [28]. Flagellin, the structural protein from the bacterial flagellum, induced the expression of the interleukin (IL)-33 receptor ST2 in the intestinal epithelium of mice co-colonized with AIEC and an attenuated strain of *S. typhimurium*, which in turn augmented IL-33 signaling and promoted the development of intestinal fibrosis [24,29].

More recently, the nuclear factor erythroid 2-related factor 2 (Nrf2)/Kelch-like ECH-associated protein 1 (Keap1) axis was suggested by Piotrowska and colleagues [19] as a promising candidate for the prevention of IBD and its severe complications, such as intestinal fibrosis, given that the Nrf2/Keap1 axis was shown to influence the production of ECM components including collagen and TGF-β1 in the gut. Notably, gut bacteria, their components (such as LPS), or their metabolites (such as urolithin A) were found to activate the Nrf2 pathway [19,30,31,32,33].

Another interesting mechanism associating intestinal fibrosis and the gut microbiome was described by Jacob and colleagues [34], who found that the intestinal fibrosis and fibroblast activation mediated by the tumor necrosis factor-like cytokine 1A (TNF-L1A) and the tumor necrosis factor ligand superfamily member 15 (TNF-SF15) are dependent on specific microbial populations and are independent of inflammation. First, they demonstrated that the profibrotic and inflammatory phenotype resulting from TNF-L1A-overexpression is abolished in the absence of resident microbiota. Then, germ-free wild-type and TNF-L1A-transgenic mice fecal transplanted (by gavage) with stools from specific pathogen-free mice and a healthy human donor showed that reconstitution with specific pathogen-free mice, but not healthy human donor microbiota, resulted in increased intestinal collagen deposition and fibroblast activation in TNF-L1A-transgenic mice. The fibrosis-triggering microbial populations were identified in the cecum as mucolytic bacteria such as the species *Mucispirillum schaedleri,* the genus *Ruminococcus*, and the genus *Anaeroplasma*, and in the ileum, such as the genera *Streptococcus* and *Lactobacillus*. In contrast, members of the genera *Oscillospira* and *Coprococcus* in the cecum, as well as *Faecalibacterium prausnitzii* and members of the genus *Bacteroides* in the ileum, were negatively correlated with fibrosis. Moreover, in vitro, some bacterial strains that were positively correlated with the degree of fibrosis promoted fibroblast migration and collagen expression, whereas other strains that were negatively-correlated with fibrosis onset did not. Interestingly, no histologically significant cecal inflammation accompanied the increased cecal collagen deposition under specific pathogen-free microbial conditions, highlighting the importance of TNF-L1A as a pro-fibrotic mediator that can act independently of its pro-inflammatory effects. In sum, this relevant study points to the existence of unique profibrotic mediators, which are either cytokine- or microbiome-driven (or both).

Corroborating the results of Jacob and colleagues [34], a cohort of children with Crohn’s disease from the RISK cohort (Risk Stratification and Identification of Immunogenetic and Microbial Markers of Rapid Disease Progression in Children with Crohn’s disease) showed that the bacteria from the genus *Ruminococcus* are implicated in structuring complications. Moreover, taxa belonging to the *Veillonella* genus were also found to be increased in penetrating complications, suggesting differential microbial populations in different disease phenotypes [35].

Beyond IBD, there is evidence as to the role of the gut microbiota in fibrosis onset in radiation-induced intestinal injury [36]. Zhao and colleagues [36] showed that antibiotic pre-treatment regimens improved the reconstitution ability of the gut microbiota in mice after radiation. This antibiotic pre-treatment in mice effectively reduced the content of LPS, inhibited the TLR4/MyD88/NF-κB signaling pathway and regulated macrophage cell polarization in the ileum, downregulated TGF-β1, phosphorylated Smad-3 and SMA protein levels and upregulated E-cadherin protein expression. In sum, Zhao and colleagues [36] suggest that antibiotic pre-treatment may significantly improve the survival rate and attenuate intestinal injury after radiation by reducing inflammation and preventing intestinal fibrosis.

In brief, there are several arguments implicating the role of the gut microbiome in the pathogenesis of intestinal fibrosis, directly or through inflammation. For that reason, modulation of the gut microbiome (as discussed in chapter 8) may constitute a valuable therapeutic tool in the management of intestinal fibrosis.

## 3. Liver Fibrosis

Liver fibrosis is an insidious process of a sustained wound-healing response to chronic liver injury, such as chronic viral infections, alcohol-related liver disease, non-alcoholic steatohepatitis (NASH) and autoimmune and genetic diseases [37]. Liver fibrosis is characterized by excessive accumulation of ECM proteins, in a dynamic process that involves complex signaling pathways and cross-talk between hepatocytes, hepatic stellate cells, sinusoidal endothelial cells and immune cells, leading to the destruction of the physiological architecture of the liver [8,37].

The gut–liver axis comprises a bidirectional interaction/communication pathway between the gastrointestinal tract and the liver, through the biliary tract, portal vein and systemic circulation, enabling the transport of gut-derived products directly to the liver, where they influence several liver functions, and the liver feedback route to the intestine, where it controls metabolic functions and influences gut barrier integrity and microbiota composition [38]. This interdependence explains the influence of the altered gut microbiome (gut dysbiosis) and disturbances in the intestinal barrier in the increased portal influx of bacteria, bacterial fragments and their products to the liver [39]. The translocated microbes and molecules then activate PPRs on liver cells, stimulating the production of inflammatory cytokines and the synthesis of ECM by hepatic stellate cells, which contribute to chronic inflammation and progressive fibrosis [40].

Recent evidence has demonstrated an association between intestinal dysbiosis and non-alcoholic fatty liver disease (NAFLD), although causality is yet to be established [41]. A change in the gut microbiota composition according to the fibrosis stage has been observed in NAFLD patients. Using 16S rRNA sequencing, Boursier et al. [42] found a higher abundance of the class Bacteroidetes and a lower abundance of the genus *Prevotella* in patients with NASH, the aggressive form of NAFLD that comprises inflammation, hepatocellular damage, steatosis and fibrosis. Among these patients (i.e., NASH) those with higher fibrosis (stage 2 or higher) also showed a higher abundance of the genus *Ruminococcus* [42]. Loomba et al. [43], using whole-genome metagenomics, identified an increased abundance of the species *Escherichia coli* and *Bacteriodes vulgatus* in patients with NAFLD with advanced fibrosis. Similarly, a greater abundance of the genus *Escherichia* has been observed in obese children with NASH, compared with children with obesity without NASH [44]. However, recently, Schwimmer et al. [45] showed that a high abundance of *Prevotella copri* was associated with more severe fibrosis in children with NAFLD. In fact, there is lack of accordance across studies, which can be explained by the great variability in study design and population selection, as well as by different geographies and dietary patterns [41]. Moreover, it is possible that the different NAFLD phenotypes may result from different microbiome signatures on the host according to its genetic predisposition or environmental factors [39].

Still, in addition to changes in the microbiome composition, alterations in the functional capacity of the gut microbiome have been demonstrated in NAFLD. When the functional profile of the gut microbiota was predicted through bioinformatics, a significant shift in the metabolic function of gut microbiota was revealed in more serious NAFLD lesions (NASH and significant fibrosis), mainly impacting Kyoto Encyclopedia of Genes and Genomes (KEGG) pathways related to carbohydrate, lipid and amino acid metabolism [42]. Moreover, in pediatric NASH patients, elevated serum ethanol concentrations have been observed, most likely from a gut-microbiota source that is enriched in alcohol-producing bacteria (e.g., *E. coli*) [44]. This may be a risk factor driving disease progression, since the role of alcohol metabolism in the generation of reactive oxygen species is well established, which then influences liver inflammation [44].

As in NAFLD, intestinal dysbiosis has been demonstrated to be an important hallmark of alcoholic liver disease (ALD). During the development and progression of ALD there are changes in the structure, composition and function of the intestinal microbiome [40]. Chronic alcohol intake causes changes in the taxonomic composition of the intestinal microbiome and increases the circulating levels of LPS, likely due to an increase in gut permeability, which then accumulate in the liver and activate PPRs, resulting, as already emphasized, in the production of inflammatory cytokines and the activation of hepatic stellate cells, which would then increase the expression of ECM [40,46]. Moreover, the severity of ALD was shown to be associated with the degree of intestinal dysbiosis [47]. Patients with severe alcoholic hepatitis harbored larger amounts of Bifidobacteria, Streptococci and Enterobacteria and less of the genus *Atopobium* compared with patients with a heavy intake of alcohol but no hepatitis [47]. Additionally, the severe phenotype was transmissible from patients to mice through fecal microbiota transplantation [47]. These animals showed an increased intestinal permeability, which led to increased bacterial translocation, along with a decrease in bile acid derivatives, which in turn could affect the efficiency of alcohol metabolism [47].

Cirrhosis, a late-stage fibrosis and an extreme manifestation of chronic liver injury, is associated with marked gut barrier impairment matching the disease progression that occurs with microbial translocation. Translocated bacteria, dominated by the phylum Proteobacteria, are abundant in the portal vein and in the hepatic and peripheral blood of decompensated cirrhotic patients and are associated with increased systemic inflammation [48]. In fact, the physiopathological mechanism involved in complications such as hepatic encephalopathy and spontaneous bacterial peritonitis is strictly associated with the translocation of enteric bacteria or their products into the systemic circulation [49]. Regarding microbiome composition, recently, metagenomic techniques have been used to characterize the fecal microbiome in cirrhosis, showing reduced diversity and overgrowth of potentially pathogenic taxa belonging to the Enterococcaceae, Staphylococcaceae and Enterobacteriaceae families, and a decreased abundance of potentially beneficial autochthonous taxa, namely, those belonging to the Lachnospiraceae and Ruminococcaceae families [50,51]. The reduced secretion of bile acid reported in cirrhosis could favor the overgrowth of these pathogenic bacteria [52]. A distinctive feature of cirrhosis is the invasion of the lower intestinal tract by microorganisms of oral origin, such as bacteria from the genera *Veillonella* and *Streptococcus* [50].

As recent evidence indicates, independently of the underlying etiology, liver fibrosis itself is typically accompanied by gut dysbiosis [53]. Together with the severely compromised gut barrier, gut dysbiosis, with the overgrowth of potentially pathogenic bacteria, drives hepatic inflammatory immune responses through portal delivery of PAMPs. These PAMPs are recognized by PPRs, such as TLRs and nucleotide-binding oligomerization domain-like receptors (NOD-NLRs), on the surface of hepatic stellate cells, hepatocytes or immune cells [54]. Therefore, hepatic stellate cell fibrogenesis can be triggered either directly or indirectly via inflammatory signals produced by neighboring cells [54]. Furthermore, the altered microbiome also results in the intestinal deconjugation of bile acids and the production of secondary bile-acids that suppress farnesoid-X receptor signaling [39]. Farnesoid-X receptor signaling exerts protective effects on intestinal epithelial barrier properties, and therefore its suppression promotes the disruption of the intestinal barrier [55], which can contribute to the perpetuation of the insult on the liver and the persistent activation of stellate cells, which would then lead to disruption of the balance between ECM deposition and dissolution, triggering progressive liver fibrosis.

In conclusion, although it is now accepted that liver damage and fibrosis can result from interplay between the gut microbiota and the host liver and immune cells, further studies are needed to better understand this interaction for future microbiome target strategies.

## 4. Kidney Fibrosis

Chronic kidney disease (CKD) is a growing global health problem, affecting at least 10 percent of the world’s population, and this percentage progressively increases with aging [56]. Fibrosis is the final pathological feature of CKD and is well recognized to contribute to the progression of almost all forms of kidney disease, behaving as an independent predictor of deterioration of renal function [57]. In CKD, fibrosis typically results from chronic inflammation of renal parenchyma [58]. Although TGF-β1 has been regarded as the main contributor to the pathological fibrotic process in CKD, metabolic and innate immune responses are now recognized as important contributors to target in this process. In addition, evidence now indicates that the activated pathological fibroblast is the dominant cell in the production of cytokines and chemokines in CKD [59].

In the last decade, an increasing amount of data has highlighted the gut microbiota as a major player in renal fibrosis, namely, by playing a relevant role in both local and systemic inflammation [60]. Several studies have clearly established that the gut microbiome population in CKD patients is significantly different from the one found in healthy subjects. Most reports are focused on end-stage renal disease (ESRD) patients and few address the gut microbiota in early CKD stages, but overall, renal disease patients present a decrease in gut microbiome diversity with a significant increase in the pathogenic bacteria of the Enterobacteriaceae family and a decrease in the beneficial microbes of the Bifidobacteriaceae and Lactobacillaceae families [61,62]. Such shifts in the gut microbiota in CKD patients from a symbiotic to a more pathogenic microbiota are well correlated with the uremic state and renal fibrosis, supporting the existence of a bidirectional and synergistic interchange between the uremic state of the host, inflammation and gut dysbiosis [63].

Progressive renal failure, in combination with the consequent changes in lifestyle, diet and medication, alters the microbiota composition and metabolism—causing dysbiosis—and the production of uremic toxins by the dysbiotic microbiota further aggravates the uremic state, damaging the epithelial barrier, increasing the intestinal permeability and promoting inflammation and oxidative stress [64]. Several in vivo and in vitro studies have shown that a uremic intestinal environment alters the tight junction protein expression pattern towards a phenotype of increased intestinal permeability [65,66,67,68], which facilitates the translocation of bacterial toxins (such as endotoxins), microbial metabolites and even bacteria into circulation [69]. DNA from intestinal bacterial populations has been detected in the blood of pre-dialysis CKD and hemodialysis patients and correlated with increased plasma c-reactive protein (CRP) and IL-6 levels [70,71,72]; gut bacterial components were found in the mesenteric lymph nodes of uremic rats [73], and endotoxemia was observed in CKD patients and correlated with systemic inflammation and cardiovascular disease in this population [74,75,76]. Bacterial translocation results in the activation of immune cells through the activation of TLR4/NF-κB/mitogen-activated protein kinases pathways, establishing a chronic inflammatory state that promotes sclerosis [76,77]. Increasing levels of circulating bacterial endotoxins across all CKD stages, reaching a maximum in dialysis patients, were found to be correlated with systemic inflammation, atherosclerosis and mortality in CKD patients [75,76]. Using an LPS-treated murine model (rats were intraperitoneally injected with 10 mg/kg/week of LPS for 4 weeks), Fereshteh Asgharzadeh and colleagues [78] showed that in clinical conditions that present chronic LPS, both cardiac and renal fibrosis can occur even in the absence of preceding tissue injury due to imbalances in oxidative stress, suggesting that gut dysbiosis may have a role in the triggering of organ fibrosis by itself.

The relevance of gut-derived circulating metabolites in the systemic immuno-inflammatory response in kidney diseases and outcomes, namely, in the associated cardiovascular risk, is now well recognized [79]. Among the different gut metabolites found to be differentially expressed in CKD patients, particular attention has been given to the effect of uremic toxins and SCFAs [79]. Higher concentrations of the uremic toxins trimethylamine N-oxide (TMAO) [80], p-cresyl sulfate (pCS) and indoxyl sulfate (IS) [81] and lower concentrations of SCFAs [82] have been consistently observed in CKD and ESRD patients [83,84]. Such results are in agreement with a report showing a significant increase in bacteria containing urease-, urase-, indole-, and para-cresol-forming enzymes and a decrease in bacteria with butyrate-forming enzymes in ESRD patients [85,86,87], further supporting a relevant contribution of gut microbiota to kidney fibrosis. 

TMAO is a gut-derived toxic metabolite that results from the bacterial metabolism of quaternary amines from the diet (such as choline, phosphatidylcholine and L-carnitine) into trimethylamine, which is then converted into TMAO by hepatic flavin monooxygenases (FMO1 and FMO3) [88]. In CKD, high TMAO levels were associated with impaired kidney function, chronic inflammation (high IL-6 and CRP), and increased mortality (a 2.8-fold increase in mortality risk) [89]. After kidney transplantation, the TMAO plasma concentration decreases to normal levels [90] but in hemodialysis patients, despite its efficient removal by hemodialysis, the TMAO plasma levels remain significantly high post-dialysis, suggesting a relevant contribution of the dysbiotic gut microbiota to the increased production of TMAO in ESRD [80,91]. Increased TMAO levels are associated with renal tubulointerstitial fibrosis and collagen deposition through the activation of the TGF-β1/p-Smad3 and renin-angiotensin aldosterone pathways [89,92]. Inhibition of TMAO production in murine models of CKD significantly retarded the decline in renal function and diminished tubulointerstitial fibrosis [93,94], highlighting TMAO production mechanisms as targets for the treatment of kidney fibrosis.

The levels of IS and pCS were found to be associated with the progression to ESRD and enhanced mortality in CKD patients from mild to severe kidney failure through enhanced oxidative stress and inflammation [81,83,95,96,97]. IS was also recently associated with CKD progression in children [98]. Increased levels of IS were shown to promote tubulointerstitial fibrosis by activating the expression of NF-κB, plasminogen activator inhibitor type 1, tissue inhibitor of metalloproteinases and TGF-β1 pathways [99]. Augmented pCS levels were associated with severe tubular damage via enhanced oxidative stress and inflammatory cytokine levels [100] and were correlated with an increased risk of all-cause mortality and cardiovascular disease in patients with chronic kidney failure and mild-to-moderate CKD [83,84]. Different animal studies showed that IS and pCS plasma concentration correlated with tubular fibrosis through activation of the intra-renal renin-angiotensin system and TGF-β1/p-Smad3 pathways [100,101]. Moreover, an increasing amount of studies have also established a relevant role of gut-derived tryptophan metabolites (namely IS, 3-IS and indole-3 acetic acid (IAA)), in renal fibrosis through the activation of aryl hydrocarbon receptor signaling pathways, specifically, through activation of aryl hydrocarbon receptor/p38 mitogen-activated protein kinase/NF-κB pathways that regulate cell proliferation, differentiation and immune function and induce cardiovascular disease in ESRD patients [102]. IAA levels have been correlated with glomerular sclerosis and interstitial fibrosis by inducing pro-inflammatory enzyme cyclooxygenase-2 and oxidative stress and its concentration was found to be predictive of mortality and cardiovascular events in patients with CKD [103].

From a different point of view, SCFAs are the major products of the fermentation of resistant starches by aerobic bacteria in the intestine [104]. Their increased concentration has been shown to exert beneficial functions in the maintenance of the epithelial barrier and in the systemic regulation of the host immune response by increasing anti-inflammatory factors, downregulating autoimmunity-related factors and developing regulatory T (Treg) cells via G-protein-coupled receptors (GPCRs) (e.g., GPR41, GPR43 and GPR109A) [105,106,107,108]. SCFAs are general histone deacetylase inhibitors that are able to inhibit TGF-β1 signaling, preventing pericyte differentiation into myofibroblasts [109,110]. Acetate, propionate and butyrate are the three most common SCFAs and constitute the primary source of energy for epithelial cells, providing approximately 10% of the daily caloric requirement in humans [104]. In kidney disease, the combinatorial effect of dietary restrictions (namely, the recommended lower intake of potassium-rich foods such as fruits, vegetables and high-fiber-containing food) and gut microbiome dysbiosis results in the reduction of SCFA-producing bacteria and in the decrease of the SCFA concentration [111]. This decreased SCFA concentration contributes to the worsening of the inflammatory processes and to kidney fibrosis [112]. An increasing amount of evidence has established SCFA supplementation as a means for the treatment of renal and cardiac disease by improving renal dysfunction, reducing local and systemic inflammation, oxidative stress and cell apoptosis [82,113].

It is now clearly established that gut microbiome dysbiosis has a significant role in kidney fibrosis and kidney disease outcomes, with several players and metabolic pathways being implicated in the process [114,115]. Still, the exact contribution of the loss of kidney function and of the dietary restrictions to this dysbiosis remains a matter of controversy. Some studies suggest that diet has the main role in the alteration of intestinal microbial metabolism observed in CKD [85], whereas others suggest that the significant gut dysbiosis observed in CKD patients is associated with increased biosynthesis of uremic toxins with a consequent impact on the decline of kidney function [116].

Multiple studies comprising prebiotics, probiotics, and symbiotics (discussed further in chapter 8) have been made, aiming to restore a healthy gut microbiota and, through this means, to manage uremia and restrain the progression of kidney diseases (characterized by increasing fibrosis of the tissue, regardless of the etiology of the disease) [117]. Despite some promising results, the highly personal response of one’s gut microbiome to multiple stimuli and the high degree of variability between the conducted studies has so far hindered the attainment of a one-size-fits-all formulation. Personalized medicine, that is, tailored approaches that address individual needs, hold great potential for the management of kidney fibrosis, which remains largely underexplored.

## 5. Pulmonary Fibrosis

Pulmonary fibrosis is a pathologic process that underlies a highly heterogeneous group of lung diseases with multiple causes, clinical manifestations and pathological features [118]. It is mainly characterized by inflammation and by the excessive deposition of ECM in the lungs, which causes architectural changes in the lung parenchyma, such as thickening and stiffening of the lung walls that may ultimately result in acute respiratory decline and organ failure [119,120]. As most pathological alterations predominate in the lung interstitium, the disorders are usually named interstitial lung diseases (ILDs). Lung damage from ILDs is often progressive and irreversible and represents an important source of morbidity and death [121].

There are more than 200 causes of ILDs. The main known triggers of the disease include age and personal genetic susceptibility (e.g., genetic conditions such as neurofibromatosis and Gaucher disease), environmental exposure to specific hazards (e.g., asbestos, silica, coal dust, beryllium, some hard metals, radiation treatments, chemotherapy drugs, antibiotics, anti-inflammatory and heart medications, animal proteins, molds or microbes) and the existence of an underlying disease such as gastrointestinal-flux disease and autoimmune diseases (e.g., lupus, rheumatoid arthritis, sarcoidosis and scleroderma) [122,123,124,125]. For management purposes, ILDs can be classified considering their known underlying diseases (e.g., pulmonary fibrosis associated with rheumatoid arthritis), on the basis of their triggering agents (e.g., pneumoconiosis), or they can be referred to as idiopathic pulmonary fibrosis [126,127]. ILDs have an overall prevalence of ~70–80 cases per 100,000 people in Europe and in the United States. Sarcoidosis, connective-tissue disease-associated ILDs and idiopathic pulmonary fibrosis are the most common fibrotic ILDs, with an estimated prevalence of 30, 12 and 8 cases per 100,000 people, respectively [128].

In recent years, several epidemiological and experimental studies have highlighted the existence of a bidirectional gut–lung axis with relevant implications in the pathophysiology of a large set of lung diseases, such as asthma, chronic obstructive pulmonary lung disease, cystic fibrosis, respiratory infections, lung cancer and also ILDs [129,130]. Multiple studies have established an intrinsic and mutual association between respiratory diseases and gastrointestinal tract diseases [131,132,133,134,135,136,137,138,139,140,141]. Considering the fact that the respiratory and the gastrointestinal tract share the same embryologic origin and are similar in structure [142], the overlap between lung and gut diseases is not surprising. The two tissues comprise an epithelial surface covered with submucosa of loose connective tissue and mucosal-associated lymphoid tissue that provide physical barriers against external microbes, regulate antigen sampling, lymphocyte transport and mucosal defense, therefore functioning as primary innate and adaptive immune responses against pathogens [142]. Moreover, both tissues are highly vascularized and colonized by microbiota that develop alongside them during early life [143].

Although most evidence regarding the role of the microbiome in the lung–gut axis remains mainly associative, some mechanistic insights support direct immunological cross-talk between the gut and the lungs, entailing the interchange of microorganisms [144,145,146,147], immune functions [17,144,146,148,149,150,151] and metabolites [129,151] through the bloodstream and the lymphatic system.

In regard to the relevance of gut metabolites in lung homeostasis, most studies show a relevant role of SCFAs in lung diseases through the inhibition of histone deacetylase and GPCRs, which are involved in lung diseases [152]. Butyrate, propionate and acetate have shown anti-inflammatory and immunomodulatory functions on lung homeostasis and immunity [104,153].

Altogether, strong evidence supports the significance of the gut microbiome and its metabolites in lung homeostasis. Taking into consideration the fact that ILDs are characterized by an enhanced inflammatory state that is similar in its players to most lung diseases, the data suggests a role of the gut microbiome in pulmonary fibrosis that remains highly underexplored. In fact, some studies report a direct link between the gut microbiome and lung fibrosis. A cross-sectional study Investigating the prevalence of intestinal dysbiosis in systemic sclerosis (a systemic connective tissue disease characterized by diffuse fibrosis and vascular abnormalities in the skin, joints and internal organs) patients showed that 76% of these patients exhibited gut dysbiosis and that the dysbiosis severity score was worse in patients with other co-morbidities, namely ILD, correlating with elevated serum inflammatory markers (e.g., CRP and erythrocyte sedimentation rate) [154].

In a study by Zhou et al. [155], the comparative analysis of the gut microbial composition of fecal samples from 18 patients with silica-induced progressive pulmonary fibrosis and 21 healthy subjects, using 16S rRNA gene sequencing technology, showed that patients with silicosis have a distinct gut microbiota profile, displaying lower levels of bacteria from the phyla Firmicutes and Actinobacteria, the genus *Devosia*, the order Clostridiales, the genus *Alloprevotella* and the Rikenellaceae RC9 gut group and an increase in taxa belonging to the Lachnospiraceae family and the *Lachnoclostridium* genus, which was correlated with disease progression.

Using animal models, Gong et al. [124] analyzed the gut microbiome and metabolites in silica and bleomycin pulmonary fibrotic models, and the combinatorial results of 16S rDNA sequencing and metabolomics supported a specific correlation between gut microbiota and pulmonary fibrosis. The retrieved results demonstrated a model-independent specific alteration in 412 genera of gut microbiota and 28 kinds of metabolites in both models. Seven representative differential gut microorganisms (from the genera *Alloprevotella*, *Dubosiella*, *Helicobacter*, *OIsenella*, *Parasutterella, Rikenella* and *Rikenllaceae RC9* group) and nine metabolites (trigonelline, betaine, cytosine, thymidine, glycerophocholine, taurocholate, adenine, deoxyadenosine, deoxycytidine) were highly correlated with fibrotic indicators. Moreover, the receiver operating characteristic curve (used to assess representative differential gut microbiota and metabolites for the diagnosing fibrotic state in the two experimental models) indicated that it is possible to distinguish proven pulmonary fibrotic status from normal control through the analysis of gut microbiota and related metabolites in mouse models [124].

Recently, a novel mouse model of scleroderma associated with anti-topoisomerase-I immunity suggested that early-life changes in the gut microbial community may shape patients’ fibrotic responses throughout their lives [156]. From a different perspective, Wand and colleagues [157] revealed that gut-lung dysbiosis-induced animal models resulted in animals with diabetes mellitus and pulmonary fibrosis associated with the NF-kB signaling pathway. 

The effects of phycocyanin (a light-harvesting protein from algal photosynthesis that can be fermented by the intestinal microbiota) on bleomycin-induced and radiation-induced pulmonary fibrosis mouse models showed that phycocyanin intervention attenuated the pulmonary fibrosis and significantly inhibited the production of inflammatory mediators, while increasing the gut bacterial diversity and richness [158,159]. In the bleomycin-induced fibrotic model, phycocyanin inhibited the synthesis of IL-1β, tumor necrosis factor-α (TNF-α), and LPSs, while significantly decreasing the bacteria related to inflammation and increasing SCFA-producing bacteria and probiotics [159]. In radiation-induced pulmonary fibrosis, both pre-administration and therapeutic administration of phycocyanin reduced inflammation damage and collagen fibber deposition and significantly reduced the levels of TNF-α, LPS and IL-6 in the lung, intestine and blood [158].

In vitro, the analysis of the effect of SCFAs on TGF-β1-induced differentiation of the MRC5 human fetal lung fibroblasts cell line showed that butyrate (C4), a SCFA that results from the metabolism of the gut microbiome, inhibits the expression of fibrosis markers and enhances mitochondrial function, therefore preventing TGF-β1-induced alveolar myofibroblast differentiation, a key factor in pulmonary fibrosis [160].

Altogether, both direct and indirect evidence implicate the gut microbiome in the regulation of the immune responses and inflammatory state of the things, with consequences relevant to the management of pulmonary fibrosis.

## 6. Heart Fibrosis

Cardiovascular diseases are a global health and financial burden, taking ~17.9 million lives every year [161]. Cardiac fibrosis is a pathological hallmark of most cardiovascular pathologies and nearly all etiologies of heart diseases involve the formation of fibrosis that persists in the myocardium of heart failure (HF) patients even after conventional treatment [162,163]. At the cellular level, fibrosis results from activation of fibroblasts by pro-inflammatory signals such as TGF-β1 [164]. In response to these stimuli, fibroblasts proliferate and differentiate in myofibroblasts, acquiring new phenotypic features such as increased expression of SMA and secretion of ECM constituents, such as collagen type I and III fibrils, which in time become cross-linked and form mature and compact fibers [165,166]. Despite being relevant to the initial repair process, namely, in the context of myocardial infarction, excessive fibrosis increases tissue stiffness, impairs cardiomyocyte coupling, facilitates arrhythmias and contributes to organ dysfunction and HF. Hence, early detection, prevention and reversion of cardiac fibrosis are key targets to advance HF management.

In the past decade, several studies have emphasized that alterations in the gut microbiome composition and intestinal permeability strongly influence the host metabolism and are associated with the development of cardiovascular disease and HF [167,168,169,170,171,172]. These studies contributed to the establishment of the “gut hypothesis of heart failure”, which proposes that dysregulation of the gut microbiome might contribute to adverse outcomes in patients with HF. In brief, a decrease in cardiac output observed in HF patients leads to a reduction in intestinal perfusion, ischemia and disruption of the mucosa, which ultimately result in higher gut permeability and alterations in gut microbial composition. Collectively, these modifications generate higher concentrations and variations in the content of microbial by-products that reach the circulation, ultimately contributing to the pathogenesis of HF. This impact may result from a direct effect of fermentation metabolites, such as TMAO, SCFAs and secondary bile acid, or indirectly, through chronic activation of inflammatory and/or oxidative stress generated by endotoxins in circulation. These molecules impact the heart by regulating several processes, including the development of cardiac fibrosis.

Cani and colleagues [173] demonstrated that gut dysbiosis, induced by means of antibiotic treatment (consisting of the reduction of *Lactobacillus* spp., *Bifidobacterium* spp. and *Bacteroides*-*Prevotella* spp.), increased systemic LPS levels and consequently increased inflammation and oxidative stress. In fact, low amounts of LPSs in the circulation trigger cardiac and renal fibrosis, even in the absence of previous injury [78,174]. Because cardiac fibroblasts express TLR4, for which LPS is a ligand, these cells directly respond to LPSs by activating the NLRP3 (NOD-, LRR- and pyrin domain-containing protein 3) inflammasome/caspase-1/IL-1β pathway, further contributing to inflammation. In fact, LPS induces IL-6 expression in cardiac fibroblasts [175], which in turn promotes the differentiation of fibroblasts in myofibroblasts and the formation of myocardial fibrosis [176]. Hence, although LPS does not directly promote fibroblast activation [177], it reprograms fibroblasts towards the formation of a pro-inflammatory and fibrogenic microenvironment in the heart.

One of the best studied gut-heart axis mediators is the microbiota-dependent metabolite TMAO that is associated with poor prognosis and increased mortality risk in cardiovascular diseases [126,178,179,180]. TMAO has also been implicated in the development of cardiac fibrosis in animal studies [181,182], namely, in the context of pressure overload [183,184]. Mechanistically, Li and colleagues [185] advanced NOD-NLRP3 inflammasome activation as a possible culprit for TMAO-mediated exacerbation of cardiac fibrosis in doxorubicin-treated mice. In fact, in vitro, TMAO induced fibroblast collagen production, proliferation and migration, suggesting that this metabolite has the capacity to directly interfere with cardiac fibroblast function [185]. Hence, the modulation of TMAO levels could be therapeutically exploited to prevent/control the formation of myocardial fibrosis. In line with this, Organ and colleagues [186] showed that TMAO levels can be reduced with either dietary withdrawal of TMAO or by blocking microbial TMAO generation using iodomethylcholine, a trimethylamine lyase inhibitor. Inhibition of TMAO by trimethylamine lyase was able to constrain the fibrotic response in response to pressure overload and a choline diet [186]. In contrast, TMAO withdrawal from the diet improved cardiac remodeling and function but no significant changes were observed in myocardial fibrosis, indicating that once established, the reversal of myocardial fibrosis is difficult to achieve [186]. The translational relevance of the fibrogenic effect of TMAO has been demonstrated in HIV patients, as TMAO was associated with diffuse myocardial fibrosis and proposed as a possible marker of early structural heart remodeling [187]. In the same study, TMAO levels were further associated with systemic levels of troponin-I, galectin-3 and N-terminal pro-brain natriuretic peptide, which have been previously associated with cardiac fibrosis.

A recent meta-analysis indicates a protective role of dietary fiber intake against cardiovascular diseases [188]. The main metabolites resulting from non-digestible fiber fermentation by gut microbiota are SCFAs, namely, acetate, butyrate and propionate, which mediate their effect by binding to GPCRs (namely, GPR41, GPR43, Olfr78 and GPR109a) and/or by inhibiting histone deacetylase activity (reviewed in [189]). Several studies have shown that SCFAs regulate blood pressure and are cardioprotective in relation to the formation of myocardial fibrosis [190,191]. In fact, GPR41, GPR43, GPR109a and GPR43/GPR109a-knockout mice develop perivascular fibrosis [192]. The same study further showed that the combination of a mild hypertensive stimulus with a diet lacking resistant starches drives gut dysbiosis and cardiac perivascular fibrosis, which was able to be rescued through the addition of acetate, propionate and butyrate to drinking water [192]. Mechanistically, Marques and colleagues [193] demonstrated that the protective effect of fiber and the SCFA acetate encompass the inhibition of cardiac early growth response-1, which has been previously associated with cardiac remodeling, inflammation and fibrosis [194]. Notably, the immunomodulatory relevance of SCFAs has also been recently highlighted, when the cardioprotective benefits of propionate, specifically in cardiac fibrosis and hypertrophy, were shown to be abrogated when regulatory T cells were depleted [195]. Although the cardioprotective effect of SCFAs seem to partially rely on a local effect on myocardial cells, is yet to be determined whether cardiac fibroblasts are responsive to these metabolites.

Secondary bile acids [196], deoxycholic acid and lithocholic acid, are a product of the microbiota of the small intestine following the processing of primary bile acids synthetized by the liver. Different studies have shown that HF and coronary artery disease patients display alterations in the excretion and serum levels of bile acid [197,198]. Overall, the structural composition and hydrophobicity of bile determine its cytotoxicity. The most hydrophilic and less cytotoxic secondary bile acid is ursodeoxycholic acid, for which beneficial effects have been reported in the heart, namely, by improving peripheral blood flow and protecting against arrhythmias [199,200,201]. Nevertheless, although ursodeoxycholic acid is able to target fibroblasts [202], the role of this secondary bile acid in the regulation of cardiac fibrosis is presently unclear [203].

Overall, the impact of the gut microbiota on cardiovascular diseases, and specifically on myocardial fibrosis, is well established. Less is known regarding the mechanisms by which the intestinal microbiota affects the heart and whether the impact on myocardial fibrosis is a direct effect on fibroblasts and/or a result of the modulation of inflammation, metabolism and/or vascular function. Advances in our mechanistic understanding of the gut-heart axis will certainly contribute to unveiling important factors for therapeutic targeting of myocardial fibrosis.

In Table 1, a summary of the main results concerning gut microbiota alterations associated with fibrosis and fibrogenic pathways in different organs is presented.

## 7. Diet as an Agent in the Development and Progression of Fibrosis

Diet has a vast impact on several aspects of our health, and a healthy lifestyle encapsulating an adequate diet and exercise routine is key to a healthy life. Inflammation is a known crucial trigger for fibrosis, and diet and inflammation are tightly linked [204,205]. For example, typical North American and Northern European diets are associated with a higher intake of saturated fats and higher levels of inflammatory markers, whereas a traditional Mediterranean dietary pattern has shown anti-inflammatory effects [205]. The gut microbiome also impacts inflammation, with a balanced bacterial community providing the necessary metabolites to inhibit intestinal inflammation [206]. A dysbiotic microbiome, on the other hand, seems to be associated with the presence of key circulating inflammatory analytes [207]. Diet plays a significant role in shaping the gut microbiome, with studies showing that dietary alterations are capable of inducing large microbial shifts [208].

Several studies associate a diet high in fat and cholesterol with different types of fibrosis (liver, lymph node and cardiac fibrosis, for example). Non-alcoholic fatty liver disease, which can progress to liver fibrosis, is characterized by excessive fatty accumulation in the hepatocytes, and the metabolic imbalance involved might occur due to increased dietary fat delivered to the liver from the gut, either due to increased intake—diet—or due to a dysregulated gut microbiome [209]. A study by Charlton and colleagues [210] reported that a “fast food diet”, a diet based on high cholesterol, high saturated fat and high fructose, was able to replicate with high fidelity the human condition of fibrosing NASH in mice, and these results were supported by Yang and colleagues [211]. Regarding lymph node fibrosis, Magnuson and colleagues [212] reported that mice fed with a high-fat diet for 13 weeks accumulated visceral fat, which was associated with increased fibrosis of the visceral lymph node. These results suggest that diet-induced visceral adiposity and inflammation can lead to immune suppression [212]. A study exploring the effects of diet on cardiac disease [213] reported that rats fed with a high-fat and high-cholesterol diet exhibited cardiac and vascular dysfunction caused by cardiac fibrosis. This research highlights how diet can lead to the development of cardiovascular disease [213].

The link between diet and fibrosis might, however, go beyond individual habits. Curiously, a study by Thompson and colleagues [214] in an animal model found that a maternal high-fat diet increased susceptibility to the development of steatosis in the offspring. Mice exposed to a perinatal and postweaning high-fat diet developed extensive hepatosteatosis compared to offspring only exposed to a postweaning high-fat diet. These results show that maternal diet can have a large role in promoting the rapid progression of NAFLD with a fibrotic phenotype [214], truly highlighting the potential of diet in fibrosis progression. Similarly to animal models, in humans, lifestyle modifications are vastly recommended in fibrotic patients, with diet-induced weight loss being a standard intervention in NASH, for example [215,216]. A low-caloric Mediterranean diet is recommended for weight loss in these patients, and several studies actually mention this diet as a protective factor from liver fibrosis [215,217]. In fact, weight loss induced by lifestyle changes is associated with the level of improvement in NASH, with fibrosis regression occurring in patients with losses of at least 10% [216,217,218].

A sufficient dietary intake, improved nutrient metabolism, supplementation with beneficial molecules (e.g., amino acids) and weight and body mass index management are critical measures to control the progression of liver and lung fibrosis [196,219,220]. Given the link between diet, inflammation and fibrosis, dietary modifications seem to be a promising strategy in fibrosis prevention and management. Likewise, the modulation of the microbiome from a dysbiotic towards a balanced state might promote the production of anti-inflammatory molecules [206], protecting against disease progression and opening up the door for the microbiome as a potential therapeutic target in fibrosis.

## 8. Modulation of the Microbiome—A Therapeutic Strategy for Fibrosis

Given the increasing evidence regarding the tight relationship between the gut microbiome and disease, the human microbiome has been gaining attention as a therapeutic target to manage or prevent a wide range of conditions [221,222]. Microbiome-targeted therapies have been proposed as a way to potentially manipulate the gut microbiome towards a desired state and therefore ameliorate dysbiosis and the associated symptoms [223,224]. These therapies generally focus on depleting overabundant species or overall microbial load using antibiotics, modulating the microbiome through dietary interventions or supplementation with pre and/or probiotics or even performing whole-microbiota transplants in the form of fecal microbiota transplantation (FMT) [221,225].

### 8.1. Antibiotic Therapy 

Antibiotics are currently used to manage chronic liver disease, which can progress to liver fibrosis [223,225]. Rifaximin, a minimally absorbable oral antibiotic, is used in the management of cirrhosis and hepatic encephalopathy, with a significant reduction in hospitalizations and an improvement in maintaining remission [223,226]. In patients with cirrhosis, rifaximin only led to minimal changes in microbial taxonomic composition, but induced significant changes in microbial metabolic function, promoting an increase in serum levels of long-chain fatty acids and carbohydrate metabolism intermediates, and a reduction in pro-inflammatory cytokine levels [223,225,227].

Regarding cardiovascular diseases, such as myocardial fibrosis, narrow-spectrum antibiotics can be used to modulate the microbiome by targeting bacteria that contribute to the production of TMAO, a cardiovascular risk predictor that is strongly associated with myocardial fibrosis [182,228]. Minocycline, for example, can reduce taxa belonging to the phylum Firmicutes, thereby ameliorating the gut dysbiosis and increasing diversity and ultimately lowering blood pressure [228,229]. However, long-term use of antibiotics may lead to the emergence of antimicrobial resistance [222,230]. With multi-drug resistant bacteria already causing much concern in our society, therapies that modulate the microbiome without drugs should be applied instead, although antibiotics can still be used in the short term before other modulation approaches (for example, before FMT or probiotic supplementation) to deplete pathogenic species and improve overall treatment efficacy [222,223,231].

### 8.2. Dietary Interventions and Prebiotic Supplementation 

The intimate connection between nutrition and the microbiome allows for the manipulation of the gut microbiota through food [232]. In fact, empirical therapeutic modulation of the gut microbiome through dietary intervention has been performed for thousands of years, for example, using traditional herbal medicines [233]. Prebiotics can be categorized as a dietary intervention, since they are defined as substrates that when consumed are selectively utilized by host microorganisms, conferring health benefits [131].

Dietary plans that are rich in prebiotics, such as the Mediterranean diet, may successfully modulate chronic kidney disease progression, protecting against kidney fibrosis [232,234]. Six months of a Mediterranean diet modulated the microbiota of CKD patients by decreasing Enterobacteriaceae and increasing some butyrate-forming species of the Lachnospiraceae, Ruminococcaceae, Prevotellaceae and Bifidobacteriaceae families [235]. Six months of a very-low-protein diet had the same effect on the microbiome, also reducing inflammatory Proteobacteria and intestinal permeability [235]. Overall, both diets decreased uremic toxins in CKD patients [235]. The ingestion of prebiotic supplements was also shown to ameliorate gut dysbiosis and CKD-associated clinical parameters, such as serum urea nitrogen, creatinine and uric acid, among others [236].

Regarding lung disease, diet fortification with fatty acids or carbohydrates of interest proved to be beneficial for lung function in cystic fibrosis patients [237,238,239]. Vitamin D supplementation could also be beneficial, since it is necessary for the development of a healthy gut microbiota in conditions defined by chronic mucosal inflammation such as cystic fibrosis [237,240].

When it comes to heart disease, a study reported that a diet high in fibers reduced blood pressure in mice with hypertension and attenuated cardiac fibrosis, showcasing the potential of dietary interventions when managing this condition [193,228].

Prebiotics can also be applied in the management of chronic liver disease. Lactulose, a prebiotic synthetic disaccharide of fructose and galactose, enhanced the growth of the *Bifidobacterium* and *Lactobacillus* genera in cirrhotic patients [241]. In fact, lactulose is already routinely used in the management of hepatic encephalopathy, with very promising results [223,225,242].

However, it should be noted that dietary interventions in a clinical context have some limitations, such as the lack of guidelines due to the need for a personalized nutritional approach, which requires both an extensive dietary analysis and the aid of a nutritionist [232]. Additionally, as with probiotics, the effects of prebiotic therapy in conditions associated with fibrosis can be mild and inconsistent, possibly due to dosage or differences in the host microbiota [221,239].

### 8.3. Probiotic and Symbiotic Therapy 

Probiotics are defined as living microorganisms which could have health benefits for the host if consumed in adequate amounts [243]. In contrast to other techniques, such as FMT, probiotic therapy constitutes a targeted modulation of the gut microbiota by adding the “healthy” probiotic to the community [221].

Several studies have shown the efficiency of probiotic treatment in ameliorating conditions that often progress to fibrosis. When reviewing the effect of probiotics on chronic liver diseases such as cirrhosis, it was found that probiotics reduced arterial ammonia concentrations, the frequency of hospitalization and the progression of hepatic encephalopathy, although larger randomized controlled trials are needed to confirm these results [223,244]. Cirrhosis patients treated with *Lactobacillus* GG for 8 weeks also experienced a reduction in dysbiosis and reduced endotoxemia and TNF-α levels compared to the placebo group [223,245]. Another review found that *Lactobacillus* GG and *Lactobacillus casei* seemed to reduce endotoxemia, dysbiosis and inflammation in cirrhosis, and a combination of *Lactobacillus* and *Bifidobacterium* species also improved liver and immune function in these patients [230,246].

In mice with chronic kidney disease, probiotic supplementation reversed the immunological alterations associated with the condition, reducing circulating levels of TNF-α and IL-6 and increasing IL-10 levels, therefore exerting a protective effect against systemic inflammation and progressive kidney fibrosis [247].

Probiotics have also shown potential in the treatment of lung disorders [239]. In patients with lung cystic fibrosis, the administration of *Lactobacillus* GG reduced pulmonary exacerbations and hospital admissions [248,249]. In children with cystic fibrosis, pulmonary exacerbations were also significantly reduced, although the effect of probiotics seems to be temporary and not permanent [250].

Regarding heart disease, in which myocardial fibrosis often develops in the final stages, probiotics formulated with *Lactobacillus* spp. have proven helpful in reducing vascular inflammation and protecting endothelial function, therefore helping to control blood pressure [228,251].

It is important to note that, although the results of probiotic therapy in fibrosis are very promising, they can also be inconsistent. These inconsistencies can be due to the dosage of the probiotic or to the strains used, which have to compete with gut pathobionts and therefore may not be able to function or survive in certain dysbiotic communities [223,239]. More research is needed in order to improve formulation and efficacy so that probiotic therapy can be a viable clinical approach for conditions associated with fibrosis in the future.

Symbiotics are a combination of probiotics and prebiotics. The prebiotics are often composed of fermented dietary fibers that aid in the growth and survival of the probiotics [223,243]. Since the prebiotics support the probiotics, this combination might promote therapeutic efficiency. A study of symbiotics in liver disease found that symbiotic treatment in cirrhotic patients increased non-urease-producing *Lactobacillus* species in the fecal content, and this modulation of the gut microbiota was associated with a reduction in blood ammonia levels and in endotoxemia [252], showcasing the potential of symbiotics in delaying the progression of liver disease.

### 8.4. Faecal Microbiota Transplantation

FMT has been used as a therapeutic tool in a range of infections and gastrointestinal conditions, and has been shown to be highly efficient in the treatment of recurrent *Clostridium difficile* infection, with an efficiency rate of over 80% [221,222,223,230,253]. In this procedure, a stool is collected from a healthy donor and transferred to a patient via a colonoscopy, nasogastric tube or enema, among other delivery routes, as a way to improve an undesired microbiome state by re-populating the gut with healthy microbiota [221,223,230,253].

Like some of the techniques already mentioned, FMT is not currently being used specifically in patients with fibrosis, although it represents a potential microbiota-targeted therapeutic strategy for associated conditions, such as NAFLD, which can progress to liver fibrosis and eventually cirrhosis [223,225], as well as viral hepatitis cirrhosis, which is characterized by diffuse fibrosis [254]. Bajaj and colleagues [255] applied FMT (a fecal suspension from a donor enriched with members of the Lachnospiraceae and Ruminococcaceae families) to 10 cirrhosis patients, following a 5-day treatment with broad-spectrum antibiotics. The results showed that FMT restored antibiotic-associated disruption in microbial diversity and function in patients with advanced cirrhosis [255].

Regarding safety, Bajaj and colleagues [256] also assessed the safety and impact of oral FMT capsules, concluding that these are safe and well tolerated in patients with cirrhosis and recurrent hepatic encephalopathy. The capsules, enriched in Lachnospiraceae and Ruminococcaceae, improved dysbiosis, duodenal mucosal diversity and duodenal antimicrobial peptide expression, and reduced serum LPS-binding protein levels [256].

Aside from chronic liver diseases, FMT is also a promising therapeutic approach in chronic kidney disease, and although there is no evidence currently available, researchers are aiming to expand its use in this field as a way to correct gut dysbiosis and protect against kidney damage, such as renal fibrosis [232].

More studies are necessary to ensure the safety and efficiency of FMT, specifically the screening of donors to avoid the spread of pathobionts to the recipient, and the standardization of sample preparation are necessary steps in order to establish FMT as a widely used, successful clinical approach [221,225,228,230,232].

## 9. Conclusions

Gut microbiome dysbiosis is emerging as a common factor across fibrotic diseases in different organs. Its established correlation with the systemic inflammatory state of the host and its emerging role in local inflammation and oxidative state are suggestive of a gut microbiome-host axis that may contribute to the regulation of fibrotic pathways underlying different diseases.

Several studies have clearly established an association between the fibrotic state of specific organs and the prevalence of pathogenic species in patients’ guts. However, results show a very high degree of variability even when addressing the same disease, hindering the translation of the acquired knowledge into clinics.

The establishment of improved sampling protocols to allow the differential analysis of different anatomic compartments of the gastrointestinal tract, in combination with holistic approaches involving high-throughput multiomics analyses to study gut–host microbiome interactions in the whole organism or in engineered microphysiological systems will provide novel insights into inter-organ communication in fibrotic diseases. These integrative approaches may lead to unprecedented understandings of genetic and epigenetic disease modifiers associated with fibrosis susceptibility and new mechanistic targets for therapy.

The modulation of gut microbial microbiota holds exciting promise for the prevention and management of fibrotic diseases, either through its direct effect in the regulation of specific fibrotic pathways or through a synergistic effect with other therapeutic options.

## Figures and Tables

**Table 1:** Alterations of gut microbiota associated with organ fibrosis.

Organ	Gut Microbiome Alterations	Possible Pathways	Mechanisms	Ref
Gut	↑ Gram-negative bacteria (↑LPS) E.g.: Adherent-invasive *Escherichia coli*; *Salmonella Enterica* Serovar *Typhimurium*. ↑ Mucolytic bacteria E.g.: *Mucispirillum schaedleri*; *Ruminococcus*; *Anaeroplasma*; *Streptococcus*; *Lactobacillus*.	-Activation of IL-33 mediated pathways; -Activation of Nrf2/Keap1 pathways; -Activation of TLR4/MyD88/NF-κB signalling pathways; -Activation of TNF-L1A (TNF-SF15) pathway.	↑ ECM deposition; ↑ Collagen expression; ↑ Fibroblast migration; ↑ Pro-inflammatory mediators; ↑ Oxidative stress mediators; ↑ Expression of profibrotic mediators (e.g.: TGF-β1, IGF-I, etc.)	[23,24,25,26,27,28,29,30,31,32,33,34,35,36]
Liver	Non-alcoholic liver diseases: ↑ Bacteroidetes ↑ *Riminococcus*, *Bacteriodes vulgatus*, *Prevotella copri* ↑ Alcohol-producing bacteria E.g.: *Escherichia coli*. ↓ *Prevotella* Alcoholic liver diseases: ↑ Enterococcaceae, Staphylococcaceae and Enterobacteriaceae ↑ Microorganisms of oral origin E.g.: *Veillonella*, *Streptococcus*. ↓ *Atopobium* ↓ Beneficial autochthonous taxa E.g.: *Lachnospiraceae*, *Ruminococcaceae*.	-Activation of hepatic inflammatory immune responses, via portal delivery of PAMPs; -Suppression of Farnesoid-X receptor signalling pathways.	≠ KEGG pathways, namely regarding carbohydrate, lipid and amino acid metabolism; ↑ Intestinal permeability; ↑ Translocation of microbes; ↑ Circulating bacterial endotoxins; ↑ ECM deposition; ↑ Pro-inflammatory mediators; ↑ Generation of reactive oxygen species; ↑Intestinal deconjugation of bile acids; ↑Production of secondary bile acids	[39,40,42,43,44,48,50,51,52]
Kidney	↓ Microbial diversity ↑ Pathogenic species E.g.: *Enterobacteriaceae*. ↓ Beneficial species E.g.: *Bifidobacteriaceae*; *Lactobacillaceae*. ↑ Urease-, urase-, indole-, and para-cresol-producing bacteria ↓Butyrate-producing bacteria	-Activation of TLR4/NF-κB/mitogen-activated protein kinases pathways; -Activation of TGF-β1/Smad pathways; -Activation of renin-angiotensin aldosterone pathway; -Activation of aryl hydrocarbon receptor signalling pathways.	↑ Intestinal permeability; ↑ Circulating bacterial endotoxins; ↑ Uremic toxins (e.g.: TMAO, pCS, IS, IAA); ↓ SCFAs; ↑ Collagen expression; ↑ Pro-inflammatory mediators (e.g.: IL-6, CRP, etc.); ↑ Oxidative stress mediators.	[61,62,64,65,66,67,68,69,70,71,72,73,74,75,76,77,78,82,83,84,85,86,87,88,102,103,104,111]
Lung	↑ Bacteroidetes, Lachnospiraceae and Lachnoclostridium ↓ *Clostridium* spp., Firmicutes, Actinobacteria, Devosia, Clostridiales, Alloprevotella and Rikenellaceae_RC9	-Activation of the TLR4/NF-kB signalling pathway.	↓ SCFAs; ↑ Pro-inflammatory mediators (e.g.: Th17 cells and IL-22).	[124,155,157,159]
Heart	↓ *Lactobacillus* spp., *Bifidobacterium* spp., *Bacteroides-Prevotella* spp.	-Activation of the NLRP3 inflammasome/caspase-1/IL-1β pathway; -Inhibition of cardiac early growth response-1.	↓ Intestinal perfusion; ↑ Collagen expression; ↑ Fibroblast migration; ↓ SCFAs; ↑ Circulating bacterial endotoxins; ↑ Microbial by-products (e.g.: TMAO); ↑ Pro-inflammatory mediators; ↑ Oxidative stress mediators; ≠ Secondary bile acids’ production.	[167,173,175,185,190,194,196]

↑ increased; ↓ decreased; ≠ altered.

## Data Availability

Not applicable.

## Abbreviations

AIEC	adherent-invasive *Escherichia coli*
ALD	alcoholic liver disease
CKD	chronic kidney disease
CRP	c-reactive protein
ECM	extracellular matrix
ESRD	end-stage-renal disease
FMO	flavin monooxygenases
FMT	fecal microbiota transplantation
GPCRs	G protein-coupled receptors
HF	heart failure
IAA	indole-3 acetic acid
IBD	inflammatory bowel disease
IGF-I	insulin-like growth factor I
IL	interleukin
ILDs	interstitial lung diseases
IS	indoxyl sulfate
Keap1	Kelch-like ECH-associated protein 1
KEGG	Kyoto Encyclopedia of Genes and Genomes
LPSs	lipopolysaccharides
NAFLD	non-alcoholic fatty liver disease
NASH	non-alcoholic steatohepatitis
NF-κB	nuclear factor-κB
NLRP3	NOD-, LRR- and pyrin domain-containing protein 3
NOD-NLRs	nucleotide-binding oligomerization domain-like receptors
Nrf2	nuclear factor erythroid 2-related factor 2
PAMPS	pathogen-associated molecular patterns
pCS	p-cresyl sulfate
PPRs	pattern recognition receptors
SCFAs	short-chain fatty acids
SMA	α-smooth muscle actin
TGF-β1	transforming growth factor-β1
TH	T helper
TLRs	Toll-like receptors
TMAO	trimethylamine N-oxide
TNF-α	tumor necrosis factor-α
TNF-L1A	tumor necrosis factor-like cytokine 1A
TNF-SF15	tumor necrosis factor ligand superfamily member 15
Treg	regulatory T cells
